# Increased Prevalence of Symptomatic Human Intestinal Spirochetosis in MSM with High-Risk Sexual Behavior in a Cohort of 165 Individuals

**DOI:** 10.3390/tropicalmed8050250

**Published:** 2023-04-26

**Authors:** Ramón Pérez-Tanoira, Marta del Palacio Tamarit, Ana María Vicente Montaña, David Carmena, Pamela Köster, Miguel Górgolas, José R. Fortes Alen, Alfonso Cabello-Úbeda, Laura Prieto-Pérez

**Affiliations:** 1 Department of Microbiology, Príncipe de Asturias University Hospital, 28805 Alcalá de Henares, Spain; 2 Department of Biomedicine and Biotechnology, University of Alcalá, 28040 Madrid, Spain; 3 Department of Internal Medicine, Fundación Jiménez Díaz University Hospital, 28040 Madrid, Spain; 4 ICTS-National Centre of Electron Microscopy, Complutense University of Madrid, 28040 Madrid, Spain; 5 Parasitology Reference and Research Laboratory, Spanish National Centre for Microbiology, Health Institute Carlos III, Majadahonda, 28040 Madrid, Spain; 6 Department of Infectious Diseases, IIS-Fundación Jiménez Díaz, 28040 Madrid, Spain; 7 Department of Pathology, IIS-Fundación Jiménez Díaz, 28040 Madrid, Spain

**Keywords:** intestinal spirochetosis, diarrhea, *Brachyspira*, MSM, chemsex, HIV infection, histopathology, metronidazole

## Abstract

Human intestinal spirochetosis (HIS) can cause gastrointestinal symptoms, although asymptomatic infections have been described. Individuals from low-income countries, people living with HIV, and men who have sex with men (MSM) show increased risk. A retrospective review of all patients diagnosed with HIS (n = 165) between January 2013 and October 2020 at a tertiary hospital in Madrid, Spain, was performed to assess risk factors for symptomatic HIS, symptoms, and response to treatment. Most patients were male (n = 156; 94.5%), 86.7% were MSM, and 23.5% practiced chemsex, of whom most were symptomatic (*p* = 0.039). Most patients (78.4%) reported unprotected oral-anal intercourse. A total of 124 (81.1%) were symptomatic; diarrhea was the most common complaint (68.3%). Multivariable regression showed increased odds of symptoms associated with age under 41 (odds ratio 5.44, 95% CI 1.87–15.88; *p* = 0.002). Colonoscopy was normal in 153 (92.7%). Furthermore, 66.7% presented previous or concomitant sexually transmitted diseases (STDs). Among the patients, 102 underwent testing for other gastrointestinal pathogens, with positive results in 20 (19.6%). All symptomatic patients without concomitant gastrointestinal infection presenting improvement on follow-up (42 of 53) had received either metronidazole or doxycycline (*p* = 0.049). HIS should be considered as a cause of chronic diarrhea in MSM with high-risk sexual behavior after other causes have been ruled out; treatment with metronidazole is recommended. Coinfection with other STDs is common.

## 1. Introduction

Human intestinal spirochetosis (HIS) is defined as the colonization of the luminal surface of colonic and appendiceal epithelial cells by anaerobic spirochetes from the genus *Brachyspira* [1,2,3]. Of the nine identified *Brachyspira* species, only *B. aalborgi* and *B. pilosicoli* are known to colonize humans [4,5,6,7].

Intestinal spirochetes were first described by Van Leeuwenhoek in 1719, who noticed moving spiral particles in his own stools, which he denominated “animalcules” [8]. In the late 19th century, during a devastating cholera epidemic in Naples, Escherich observed spirochetes in the stools of both symptomatic and asymptomatic individuals [9]. The term HIS was coined in 1967 by Harland and Lee to describe the intestinal colonization of a 64-year-old male patient presenting with a 3-year history of chronic diarrhea [1]. Since then, numerous cases of HIS from low, middle, and high-income countries have been reported.

Spirochetes are a well-known cause of diarrhea in animals [10,11,12,13,14] and have been isolated from the feces of birds, pigs, dogs, and primates. In developing countries, contaminated water and infected animals are primary sources of HIS, whereas, in developed countries, homosexual intercourse is a common risk factor [15,16,17,18,19].

The pathogenic ability of spirochetes is still under debate, with some authors considering HIS harmless [20]. However, a recent systematic review and meta-analysis found that HIS was significantly associated with diarrhea and abdominal pain [7]. Van Mook et al. suggest that, occasionally, the microorganism may gain pathogenicity and become invasive [17,20,21]. The clinical spectrum of HIS is broad, ranging from asymptomatic colonization and incidental diagnosis to symptoms such as abdominal pain, meteorism, diarrhea, constipation, or bleeding. When symptomatic, HIS usually presents as chronic, watery diarrhea [22].

HIS has traditionally been associated with immunosuppression [23,24], especially in patients with HIV infection, though it may also occur in patients with a normal immune status [7,25]. Previous reports suggest that HIS prevalence is significantly higher amongst men who have sex with men (MSM), leading to the question of whether HIS should be considered a sexually transmitted disease (STD) [26]. Individuals at high risk for STDs include those having unprotected sexual intercourse. Those participating in sexualized drug use or chemsex, the use of substances (typically crystal methamphetamine, mephedrone, GHB/GBL, and ketamine) to facilitate, disinhibit, prolong and/or intensify sexual experience [27] (mainly MSM) are at an increased risk for STDs, due to a lower perception of risk and a subsequent increase in unprotected sexual intercourse.

A diagnosis of HIS is made by routine hematoxylin and eosin staining of colonic mucosal biopsies, revealing a dense, bluish haze on the luminal surface of enterocytes caused by a palisade-like arrangement of bacteria that gives the impression of a tight ‘false brush border’ [21]. Subsequent confirmation with Warthin-Starry or Dieterle silver impregnation is highly recommended, as is immunochemistry.

Treatment is not recommended in asymptomatic patients, and a wait-and-see attitude may be followed [21]. However, metronidazole has been described as an effective treatment of anaerobic spirochetes in patients presenting with chronic diarrhea [7,28,29].

The aim of this study was to analyze the clinical significance of HIS in patients from a tertiary hospital in Spain by investigating possible modes of transmission, risk factors for symptomatic disease, and describing histological features and response to treatment.

## 2. Materials and Methods

We performed a retrospective study of all cases of HIS diagnosed between 1 January 2013 and 31 October 2020 at the Fundación Jiménez Díaz University Hospital in Madrid (Spain). This study included 165 consecutive colorectal biopsies.

### 2.1. Study Population and Sample Collection

All consecutive cases in which spirochetes were visualized in colonic biopsies obtained from colonoscopies performed from January 2013 until October 2020 were included. A colonoscopy was performed to study symptomatic patients presenting with abdominal pain, chronic diarrhea, or bleeding. Colonoscopies were performed in asymptomatic individuals as the screening procedure in patients with a family history of colon cancer; furthermore, biopsies were also obtained in macroscopically normal colonoscopies in order to rule out microscopic colitis such as lymphocytic and collagenous colitis. Biopsy specimens were obtained from the colon and rectum and evaluated histologically at the Department of Pathology at the Fundación Jiménez Díaz University Hospital in Madrid (Spain). HIS was diagnosed using light microscopy of colorectal mucosa biopsies. The identification of a 3 µm hematoxylinophilic fringe formation adjacent to the normal brush border of the luminal side of the enterocytes was considered a positive result. This finding is proof of spirochete colonization of the colon. The stepped biopsy specimens were fixed in 10% formalin, dehydrated in an increasing series of alcohols and xylol, and then embedded in paraffin. After being deparaffinized, the 4 mm-thick stepped sections (at least 8 sections per block) were routinely stained with hematoxylin and eosin. Diagnoses were confirmed with Warthin-Starry silver stain, and in selected cases, immunohistochemistry with anti-*Treponema pallidum* antibodies was also performed (rabbit polyclonal antibody Ref: AP10661; AP10661C. Gennova Scientific, S.L. Seville, Spain).

Informed consent was obtained from all subjects involved in the study. Clinical and epidemiological data were retrospectively collected from the medical records of patients to whom a standardized questionnaire had previously been administered. However, some data are missing either because they were not collected at admission or because the patient did not answer or consent to the recording of some personal information; therefore, the denominators for the different characteristics vary. Information about endoscopic findings, treatment, and post-treatment symptoms was also obtained from clinical records. This included: (i) demographic characteristics, e.g., age, sex, and country of birth, (ii) behavioral habits, e.g., contact with animals, travel to developing countries, hand and fruit/vegetable washing, and whether there has been any occurrence of diarrhea in the participant or their family members (iii) drinking and recreational water use, e.g., type of drinking water, whether they had swum in pools, and (iv) sexual attitudes, e.g., sex orientation, sex-role preference, sex-risk behavior, and sexualized drug use (chemsex).

### 2.2. Scanning Electron Microscopy

In one case, an additional electron microscopic examination was carried out (JEOL JSM6400 scanning microscope, Tokyo, Japan). Scanning Electron Microscopy (SEM) was retrospectively performed on a formalin-fixed large bowel sample from a colonoscopic biopsy. Tissue was processed according to the conventional SEM protocol of fixation with 2% glutaraldehyde and 4% paraformaldehyde in Millonig’s buffer solution at room temperature for one hour, followed by waxing in distilled water and subsequent dehydration with a series of increasing ethanol concentrations. Millonig buffer consists of 2.26% sodium phosphate dibasic solution, and it was laboratory-made with 2.26 g NaH_2_PO_4_ + 100 mL of double-distilled water; 2.52% sodium hydroxide solution (2.52 g NaOH + 100 mL of double-distilled water) and 5.4% glucose solution (5.4 g C_6_H_12_O_6_ + 100 mL of double-distilled water). The solution is 0.1 M, and its pH 7.3. Fixation with glutaraldehyde (25% solution EM Sciences, Hatfield, PA, USA) and paraformaldehyde (16% solution EM Sciences, Hatfield, PA, USA) result in Karnovsky fixer. This fixer is preferred above others since bacterial coating and culture features are better preserved. The dehydration process was completed using a critical point dryer. Finally, the sample was mounted in a holder for observation with a JEOL JSM6400 scanning microscope at 20 kV.

### 2.3. Microbiological Studies

Patients diagnosed with HIS were screened for concomitant STDs and gastrointestinal infections.

#### 2.3.1. Sexually Transmitted Diseases

STDs included sexually acquired HIV, hepatitis C, hepatitis B, and syphilis. Additionally, urethritis and proctitis were studied and defined as the presence of one or more signs or symptoms as depicted in specific guidelines: dysuria, urethral pruritis, mucoid, mucopurulent, and purulent discharge for urethritis and rectal bleeding, pain, tenesmus, constipation, and anal discharge for proctitis [30,31].

*Chlamydia trachomatis* was detected by polymerase chain reaction on urethral or rectal swab specimens. The swabs used for sample collections were CE medical devices class IIa, DELTALAB (Barcelona, Spain). We used multiplex polymerase chain reaction (*C. trachomatis*/*Neisseria gonorrhoeae*/*Mycoplasma genitalium* Real-TM; Sacace Biotechnologies Srl, Como, Italy) to detect *C. trachomatis* infection by targeting an 89-bp region of the cryptic plasmid and a 162- to 165-bp region in the genome. The identification of serovar and serogroup was made by specific probes (RHA kit Ct Genotyping, Labo Biomedical Products BV, Rijswijk, The Netherlands); since performing this test takes up to 5 h and because of the large number of samples received in our laboratory, the result could be delayed a few days. When performing all tests, the manufacturer’s instructions were strictly followed.

Urethral or rectal swabs for *N. gonorrhoeae* culture on selective media, other selective media for facultative bacteria, and polymerase chain reaction to detect herpes simplex virus (artus^R^ Herpes simplex virus 1/2 QS-RGQ; Qiagen, Hilden, Germany) were also performed. Likewise, we also carried out blood tests to detect other STDs, such as HIV, syphilis, hepatitis B, and hepatitis C serologic tests.

#### 2.3.2. Gastrointestinal Infections

From 2013 to 2020, fecal samples were processed using standard microbiological culture methods, namely bacterial culture, microscopy examination for parasites, and immunochromatography for adenovirus, rotavirus, or norovirus. Furthermore, molecular detection of protist intestinal parasites was performed in samples with a positive result on direct microscopy, and the BioFire^®^FilmArray^®^ gastrointestinal (GI) (Biomerieux, Marcy l’Étoile, France) panel was only carried out when requested by the clinician.

- Bacterial culture

Fecal samples were cultured on CCDA agar plates (Oxoid, Basingstoke, UK) for *Campylobacter* isolation, SS agar plates (Becton Dickinson, Heidelberg, Germany) for *Shigella* and *Salmonella* spp., and CIN agar plates (Oxoid) for *Yersinia*. In order to detect oxidase-positive Gram-negative bacilli, such as *Vibrio*, *Plesiomonas*, and *Aeromonas*, an oxidase test was performed on the bacterial colonies grown on blood agar (Oxoid, Basingstoke, UK) and MacConkey agar (Becton Dickinson, Heidelberg, Germany) [32]. A Rappaport–Vassiliadis *Salmonella* Enrichment Broth (VWK Chemicals, MerckKGaA, Darmstadt, Germany) was used as an enrichment step for the recovery of *Salmonella*, followed by plating on SS agar. The final identification was achieved using matrix-assisted laser desorption/ionization time-of-flight mass spectrometry (MALDI-TOF) (Bruker, Bremen, Germany) following the manufacturer’s instructions [32].

- Direct microscopy

Wet mounts for direct microscopic observation of fresh stools were performed at the Department of Microbiology of Fundación Jiménez Díaz University Hospital. When *Entamoeba histolytica* was suspected, stool samples were periodically sent to the Reference and Parasitological Research Laboratory of the National Microbiology Centre (Instituto de Salud Carlos III, Madrid, Spain) for PCR techniques to differentiate it from *Entamoeba dispar*, a morphologically identical non-pathogenic species on direct microscopy [33].

- Molecular detection

Genomic DNA was isolated from about 200 mg of each fecal specimen of wild ungulate origin by using the QIAamp DNA Stool Mini Kit (Qiagen, Hilden, Germany) according to the manufacturer’s instructions, except for samples mixed with InhibitEX buffer that were incubated for 10 min at 95 °C. Extracted and purified DNA samples were eluted in 200 µL of PCR-grade water and kept at 4 °C until further molecular analysis.

Detection and differential diagnosis of *E. histolytica* and *E. dispar* were carried out by a qPCR method targeting a 172-bp fragment of the *ssu* rRNA gene of the *E. histolytica*/*E. dispar* complex using the generic primer set Ehd-239F/Ehd-88R [34]. All qPCR reactions were run on a Corbett Rotor-Gene 6000 qPCR cycler (QIAGEN). All direct and nested PCR reactions were run on a 2720 thermocycler (Applied Biosystems, Foster City, CA, USA). Reaction mixtures included 2.5 units of MyTAQ™ DNA polymerase (Bioline GmbH, Luckenwalde, Germany), a 5xMyTAQ Reaction Buffer containing 5 mM dNTPs, and 15 mM MgCl_2_. PCR amplicons were visualized on 2% D5 agarose gels (Conda, Madrid, Spain) and stained with Pronasafe nucleic acid staining solution (Conda).

When requested by the clinician, assays on the feces samples were performed using the Biofire^®^FilmArray^®^ gastrointestinal panel for the detection of 22 pathogens, which are causative agents of infectious diarrhea in humans, including bacteria, *Campylobacter* (*jejuni*, *coli*, *upsaliensis*), *Clostridioides difficile* toxin A/B, *Plesiomonas shigelloides*, *Salmonella*, *Yersinia enterocolitica*, *Vibrio* (*parahaemolyticus*, *vulnificus*, *cholerae*), *Escherichia coli* O157, *enteroaggregative E.coli* (EAEC), *enteropathogenic E. coli* (EPEC), *enterotoxigenic E. coli* (ETEC) Shiga toxin/verotoxin-producing or *enterohaemorrhagic E. coli* (STEC) stx1/stx2, and *Shigella/Enteroinvasive E. coli* (EIEC); parasites, *Cryptosporidium*, *Cyclospora cayetanensis*, *Entamoeba histolytica*, and *Giardia lamblia*; and viruses including Adenovirus F 40/41, Astrovirus, Norovirus GI/GII, Rotavirus A, and Sapovirus(I, II, IV, and V). For each sample, 200 µL was added to each panel, following the manufacturer’s instructions, and was then analyzed in a Biofire^®^Filmarray^®^ integrated system (Biomerieux, Marcy l’Étoile, France).

### 2.4. Statistical Analysis

Continuous variables were presented as median and interquartile ranges (IQR), and categorical variables as proportions unless otherwise specified. We used the Mann-Whitney U-test, χ^2^ test, or Fisher’s exact test to compare differences between groups, as appropriate. For these comparisons, a *p*-value of 0.05 or lower was considered of statistical significance. Statistical analysis was performed using SPSS^®^ v27.0 (IBM Corp., Armonk, NY, USA).

Univariable and multivariable logistic regression models served to analyze risk factors associated with symptoms. Values are expressed as odds ratio (OR) and 95% confidence intervals (95% CI). The multivariable logistic regression model was adjusted by the variables that had *p*-value ≤ 0.05, which were further selected by a stepwise forward selection method (pin < 0.05 and pout < 0.10). Significant differences are shown in bold.

## 3. Results

### 3.1. Age and Sex

A total of 165 patients were diagnosed with HIS, with a median age of 41.0 years (IQR 34.0–50.0). Most patients were male (156/165; 94.5%), and the majority were Caucasian (148/165; 89.7%). The baseline characteristics of the patients are shown in Table 1.

### 3.2. Lifestyle and Risk Factors

A total of 99 patients were smokers (99/165; 60%), 36 patients acknowledged using one or more recreational drugs (36/119; 30.2%), and 27 (27/115; 23.5%) engaged in chemsex. The most common recreational drugs were GHB, mephedrone, popper, crystal methamphetamine, and, to a lesser extent, ketamine.

Regarding other potential risk factors for HIS, 67 patients (67/106; 63.2%) had traveled to low and middle-income countries in the past, and 61 patients (61/101; 60.4%) reported previous or current animal ownership.

### 3.3. Sexual Orientation, HIV Status, and Sexually Transmitted Diseases

Data regarding sexual orientation and drug use were collected from patients who agreed to share this information; the sample size of each variable is specified in Table 1. HIV was diagnosed after HIS was detected. In the male group, 124 patients reported having sex with other men (124/143; 86.7%); 85 patients did not have a stable sexual partner (85/125; 68.0%), and 98 patients reported habitual, unprotected oral-anal contacts (98/125; 78.4%).

Information on HIV testing was only available for 153 patients, of whom 65 (42.5%) were HIV-positive. In one case, HIV was diagnosed after HIS was detected. During the period of study, 98 patients (98/147; 66.7%) presented a concomitant STD. A total of 12 patients (12/147; 8.2%) had a previous diagnosis of sexually acquired hepatitis C, 4 tested positive for hepatitis B surface antigen, and 47 (47/141; 33.3%) for a positive core antigen (HBcAg). Additionally, 44 patients (44/127; 34.6%) had a positive treponemal test. Moreover, 43 patients (43/103; 41.7%) had presented a previous episode of urethritis, most commonly caused by *Neisseria gonorrhoeae*, and 33 patients (33/99; 33.3%) proctitis, mainly caused by *Chlamydia trachomatis* (Table 2).

### 3.4. Symptomatology

Gastrointestinal symptoms were recorded in 133 patients (81.1%); 112 patients (68.3%) presented with diarrhea, 79 patients complained of changes in their bowel habits (48.2%), 51 had experienced rectal bleeding (31.1%), and 49 abdominal pain (29.9%).

Risk factors associated with the presence of symptomatic disease are shown in Table 3. Multivariable regression showed an increased odds of symptoms associated with age under 41 (odds ratio 5.44, 95% CI 1.87–15.88; *p* = 0.002).

### 3.5. Localization and Histologic Findings

**Figure 1:**
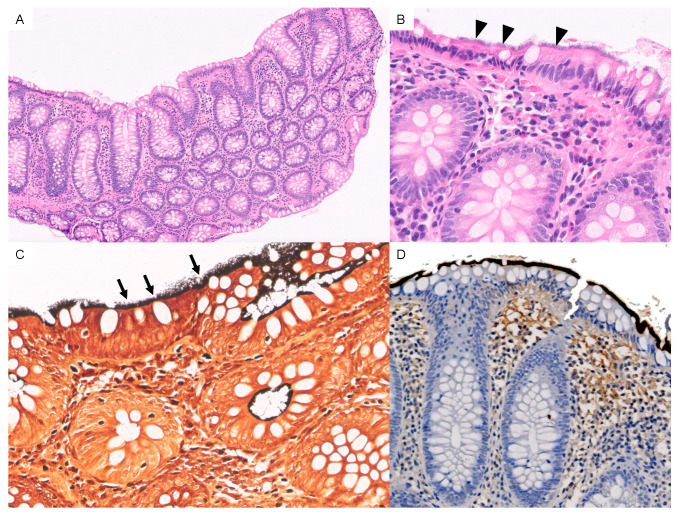
(**A**) Panoramic view of HIS with hematoxylin and eosin staining [10×]. (**B**) A 3 μm-thick basophilic fringe on the luminal surface of the enterocytes is visible (arrowheads) [40×]. (**C**) Spirochetes with Warthin-starry silver stain are observed (arrows). [40×] (**D**) Immunohistochemistry for anti-*Treponema pallidum* antibodies highlights spirochetes in dark brown-black color.

Macroscopic colonoscopy findings were normal in 152 patients (92.1%). Unspecific inflammatory changes were observed in the remaining 7.9%. HIS was mainly detected in the cecum and ascending colon in 128 patients (77.6%), followed by the transverse colon (50.9%), descending colon (44.2%), and rectum (26.1%). Biopsies showed spirochetes in a variety of localizations: on the intact surface epithelium (n = 152; 92.1%); in the upper part of crypts (n = 9; 5.5%); on the surface of microabscesses (n = 2, 1.2%); and in both the upper part of crypts and microabscesses (n = 1, 0.6%). Spirochetes were found coating adenomas in 25 samples (15.15%). Under light microscopy, spirochetes were observed attached to the surface of the colonic epithelium with hematoxylin-eosin staining. Warthin-Starry silver staining was performed in all biopsies, confirming the presence of HIS (Figure 1).

Additionally, immunochemistry for anti-*Treponema pallidum* antibodies was carried out in some samples (Figure 1D).

#### Scanning Electron Microscopy (SEM)

**Figure 2:**
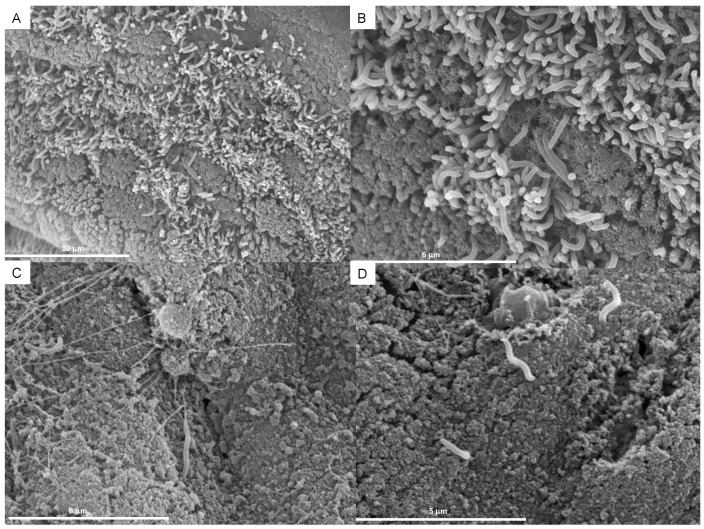
(**A**) Spirochetes attached to the colonic epithelium. (**B**) Detail of spirochetes disrupting the normal architecture of the human colon, invading the epithelium. (**C**) Biofilm formation of *Brachyspira aalborgi*. (**D**) Detail of spirochetes with their distinctive sinusoidal shape.

One specimen was obtained for study under SEM, where the colonic epithelium was found to be covered with a forest of spirochetes. *B. aalborgi* was identified using polymerase chain reaction (PCR). Figure 2 shows how the microorganism disrupts the normal architecture of the human colon, invading the epithelium and creating a biofilm.

### 3.6. Additional Microbiological Studies

In total, 102 fecal samples from symptomatic patients were sent for microbiological testing. Among them, 20 (19.6%) were positive for an infective microorganism (Table 4), and seven patients were coinfected with more than one pathogen. The most common microorganism was *Giardia duodenalis* (n = 8), followed by EAEC (n = 5), Shigella/EIEC (n = 3), EPEC (n = 3), STEC (n = 2), *Blastocystis hominis* (n = 2), *Entamoeba hystolitica* (n = 1), Norovirus (n = 1), Sapovirus (n = 1) and *Yersinia enterocolitica* (n = 1). It was necessary to perform molecular differentiation between *E. hystolitica* and *E. dispar* in 14 cases. In only one patient, *E. hystolitica* was detected, while *Entamoeba dispar* DNA was detected in all of the others (92.8%). The results of pathogenic microorganisms are shown in Table 4.

### 3.7. Blood Tests

Blood test results showed no specific findings and were otherwise unremarkable (Table 5).

No statistically significant difference was found when comparing these values between symptomatic and asymptomatic patients.

### 3.8. Treatment

Out of 165 patients, 107 (69.0%) were treated. Additionally, 91 (58.7%) received metronidazole, 12 were treated with doxycycline (7.3%), and the four remaining patients received different antimicrobial agents (macrolides or combined treatment).

Of the 82 symptomatic patients without concomitant gastrointestinal infection, 53 followed up after treatment; all patients reporting symptom improvement (n = 42, 79.2%) had received metronidazole or doxycycline (*p* = 0.049).

## 4. Discussion

HIS is caused by the slow-growing anaerobic gram-negative spirochetes *B. aalborgi* and *B. pilosicoli*. Previous descriptions of HIS report an association with HIV infection and MSM, but various studies have confirmed that it also affects a wider range of patients, including those living in relatively crowded conditions, lacking proper hygienic facilities, and living in close contact with animals [1,15,17,19].

Every patient in whom HIS was incidentally found during a colonoscopy was selected. Colonoscopies were performed irrespective of the patient’s sexual orientation, finding the vast majority of HIS in MSM as in other studies [4,7,15,16,17,18,19,35,36]. The age profile of our cohort is similar to that of prior publications [6,37]. HIS has traditionally been associated with HIV infection, and patients with a new diagnosis of HIS should be screened for HIV infection. In our series, one new HIV diagnosis was made after screening in the context of HIS.

We observed a high *N. gonorrhoeae* and *C. trachomatis* coinfection rate, a finding reported in other studies [19,26]. *Giardia duodenalis*, *Entamoeba histolytica*, and other intestinal protozoa cause symptoms resembling those of symptomatic HIS; metronidazole is the treatment of choice for these pathogens, but it is also first-line therapy for HIS. Screening for intestinal protozoa and appropriate treatment may be considered as a first step when evaluating patients with possible HIS to minimize confounding factors and avoid unnecessary invasive procedures.

In our series, coinfection with another gastrointestinal pathogen was detected in 20 symptomatic patients (19.6%), with HIS being the sole infectious agent in 80.4%. All symptomatic patients without a concomitant gastrointestinal infection showing improvement at the follow-up had received treatment with metronidazole or doxycycline.

To date, and to the best of our knowledge, no previous studies have assessed the role of sexualized drug use in HIS. A vast majority of HIS was detected in MSM (86.7%), among whom 23.5% practiced chemsex. Although a higher number of chemsex users was found in symptomatic compared to asymptomatic HIS, chemsex was not confirmed in the multivariate analysis as an independent risk factor for the development of symptoms, being rather a co-factor that increases rates of unprotected sex, leading to an increased risk of STDs [38,39].

The absence of macroscopical inflammation in our series contrasts with the findings of Calderaro et al., who describe hyperemia, mucosal erosion, and inflammatory changes in the 17 patients included in their study [36]. However, our results are similar to those described by Anthony et al., who found no macroscopical inflammation or, at most, minimal inflammatory changes [6].

Therapeutic recommendations for HIS have changed over the last decades, and response to treatment varies. Some patients may experience complete remission of diarrhea and gastrointestinal symptoms, whereas others fail to improve despite confirmation of HIS eradication in subsequent histologic exams [22]. Some authors suggest that the level of histological invasion could be associated with symptom severity, with patients who present spirochaetal invasion beyond the superficial epithelium showing better response to treatment [40]. In symptomatic patients, a trial of antibiotic treatment with metronidazole is recommended [7,28,29,41], although clinical responses may vary. Metronidazole is one of the mainstay drugs for the treatment of anaerobic infections; it exerts rapid bactericidal effects against anaerobic bacteria with a killing rate proportional to the drug concentration. The most common adverse effects are gastrointestinal, and patients may present with symptoms such as nausea, anorexia, vomiting, and diarrhea. It can also cause dizziness, confusion and peripheral neuropathy, and a disulfiram-like reaction may be observed in patients drinking ethanol [42].

Doxycycline, in turn, inhibits protein synthesis by binding with the 30S ribosomal subunit of susceptible bacteria. It may cause skin photosensitivity reactions, and also esophagitis and esophageal ulcers may occur due to local caustic injury produced by direct local contact and because of its low pH. In our study, we found that all symptomatic patients without concomitant gastrointestinal infection improved after being treated with metronidazole or doxycycline. No adverse reactions were reported in either case.

Our histopathological findings of HIS using light microscopy are similar to those described in the literature. We only recruited one sample for scanning electron microscopy, which showed a disruption of the normal architecture of the human colon and the presence of biofilm. Recent studies have demonstrated the capability of some spirochetes to form biofilms, as in the case of *Borrelia burgdorferi*, *Leptospira interrogans*, and *Treponema denticola* [43,44]. This is the first report of biofilm formation in *Brachyspira* spp, although the finding should be confirmed in future studies.

The main limitation of this study is its retrospective nature, so the statistical analysis was most likely influenced by the limited availability of some data. As reported in the methods, clinical and epidemiological data were retrospectively collected from the medical records of patients to whom a standardized questionnaire had previously been administered. PCR in fecal samples was performed irrespective of symptomatology, but data were not available for all samples. Moreover, molecular studies to identify the species of *Brachyspira* were only performed in one case.

## 5. Conclusions

HIS should be considered as a cause of chronic diarrhea in MSM with high-risk sexual behavior after other causes have been ruled out. Treatment with metronidazole is recommended in these cases. Coinfection with other sexually transmitted diseases is common.

## Figures and Tables

**Table 1:** Sociodemographic variables and association with symptomatic HIS.

	Total	Symptomatic n = 133	Asymptomatic n = 31	*p*-Value
Age (median)	41.0 (IQR:34.0–50.0)	39 (IQR:33.0–48.0)	50 (IQR:43.0–57.0)	**<0.001**
Male (n = 165)	156 (94.5%)	127 (95.5%)	28 (90.3%)	0.255
Spanish nationality * (n = 164)	135 (82.3%)	109 (82.6%)	25 (80.6%)	0.800
MSM (n = 143)	124 (86.7%)	102 (87.9%)	21 (80.8%)	0.332
No steady partner (n = 125)	85 (68.0%)	70 (69.3%)	15 (62.5%)	0.627
HIV+ (n = 153)	65 (42.5%)	55 (44.4%)	10 (34.5%)	0.333
Alcohol (n = 142)	83 (58.5%)	70 (60.3%)	13(50.0%)	0.333
Drug use (n = 119)	36 (30.2%)	33 (34.7%)	3 (12.5%)	**0.034**
Sexualized drug use (chemsex) (n = 115)	27 (23.5%)	25 (27.8%)	2 (8.0%)	**0.039**
Prior travel to developing countries (n = 106)	67 (63.2%)	52 (61.9%)	15 (68.2%)	0.587
Animal ownership (n = 101)	61 (60.4%)	44 (56.4%)	17 (73.9%)	0.131

MSM, Men who have sex with men. * Other nationalities: Lebanon, Greece, Dominican Republic, Philippines, Morocco, Belgium, Poland, Chile, Bolivia, and Colombia. In one patient, the presence of symptoms was not available in the medical record. *p*-values < 0.05 are shown in black.

**Table 2:** Sexually transmitted diseases in MSM patients with HIS.

	n (%)
Urethritis n = 103	43 (41.7%)
*Neisseria gonorrhoeae*	36 (35.0%)
*Chlamydia trachomatis*	11 (10.7%)
*Treponema pallidum*	3 (2.9%)
Herpes simplex 1–2	1 (1.0%)
*Ureaplasma urealyticum/* *Mycoplasma genitalum*	5 (4.9%)
Proctitis n = 99	33(33.3%)
*Neisseria gonorrhoeae*	9 (9.1%)
*Chlamydia trachomatis*	24 (24.2%)
*Treponema pallidum*	2 (2.0%)
Herpes simplex 1–2	5 (5.1%)
*Ureaplasma urealyticum/* *Mycoplasma genitalum*	8 (8.1%)

**Table 3:** Risk factors associated with symptomatic HIS.

	Univariate OR (95% CI)	*p*-Value	Multivariate OR (95% CI)	*p*-Value
Age < 41 years	5.39 (2.07–14.00)	**≤0.001**	5.44 (1.87–15.88)	**0.002**
Male (n = 165)	2.27 (0.54–9.62)	**0.267**		
Spanish nationality * (n = 164)	1.14 (0.42–3.09)	0.800		
MSM (n = 143)	1.74 (0.56–5.34)	0.337		
No steady partner (n = 125)	1.36 (0.535–3.43)	0.521		
HIV+ (n = 153)	1.51 (0.65–3.52)	0.335		
Alcohol (n = 142)	1.52 (0.65–3.58)	0.335		
Drug use (n = 119)	3.73 (1.04–13.42)	**0.044**	Not included *p* > 0.05	n.s.
Sexualized drug use (chemsex) (n = 115)	4.42 (0.97–20.16)	**0.055**	Not included *p* > 0.05	n.s.
Prior travel to developing countries (n = 106)	0.76 (0.28–2.06)	0.587		
Animal ownership (n = 101)	0.46 (0.16–1.28)	**0.137**		
STD (n = 147)	0.87 (0.35–2.16)	0.760		
DPS (n = 102)	0.79 (0.20–3.11)	0.731		

MSM, Men who have sex with men; DPS, Detected Pathogen in Stools; STD, Sexually Transmitted Diseases. * Other nationalities: Lebanon, Greece, Dominican Republic, Philippines, Morocco, Belgium, Poland, Chile, Bolivia, and Colombia. In one patient, the presence of symptoms was not available in the medical record. *p*-values < 0.05 are shown in black.

**Table 4:** Results of microbiological studies in stool samples from 102 patients.

Microorganism	n
*Giardia duodenalis*	5
EAEC	2
*Campylobacter* sp.	2
*Blastocystis hominis*	2
EPEC	1
STEC	1
*Campylobacter* sp. and *Shigella*/EIEC	1
*Campylobacter* sp., EAEC, EPEC and *Shigella*/EIEC	1
*Giardia duodenalis*, *Campylobacter* sp. and EPEC	1
*Giardia duodenalis*, *Cryptosporidium* spp., *Entamoeba hystolica* and EAEC	1
Shigella/EIECand Norovirus	1
Sapovirus and EAEC	1
STEC, *Yersinia enterocolitica* and *Giardia duodenalis*	1
Negative	82

Enteropathogenic (EPEC), enteroinvasive (EIEC), enteroaggregative (EAEC), and Shiga toxin/verotoxin-producing or enterohaemorrhagic (STEC) *Escherichia coli*.

**Table 5:** Results of blood tests.

	Leucocyte (cel/mm^3^)	Hb (g/dL)	Platelets (cel/mm^3^)	Creatinine (mg/dL)	C-Reactive Protein (mg/dL)
Median	7050.00	15.1	232,000	0.90	0.36
IQR	6040.00–8510.0	14.5–15.8	203,000–283,000	0.80–1	0.11–1

## Data Availability

Data cannot be shared publicly because they are confidential. Data are available from the Department of Infectious Diseases of IIS-Fundación Jiménez Díaz for researchers who meet the criteria for access to confidential data.
